# Pain assessment and factors influencing pain during bone marrow aspiration: A prospective study

**DOI:** 10.1371/journal.pone.0221534

**Published:** 2019-08-29

**Authors:** Nicolas Gendron, Sara Zia Chahabi, Géraldine Poenou, Nadia Rivet, Tiphaine Belleville-Rolland, Pierre Lemaire, Antoine Escuret, Michèle Ciaudo, Emmanuel Curis, Pascale Gaussem, Virginie Siguret, Luc Darnige

**Affiliations:** 1 AP-HP, Hôpital Européen Georges Pompidou, Service d’Hématologie Biologique, Paris, France; 2 Université de Paris, Paris, France; 3 INSERM UMR-S1140, Paris, France; 4 AP-HP, Hôpital Lariboisière, Paris, France; 5 Laboratoire de Biomathématiques, plateau *i*B^2^, Faculté de Pharmacie de Paris, Université Paris Descartes, Paris, France; 6 Service de Biostatistiques et Informatique Médicale, AP-HP, Hôpital Saint-Louis, Paris, France; Cleveland Clinic, UNITED STATES

## Abstract

Although bone marrow aspiration (BMA) is still considered a painful procedure, pain level remains poorly documented. We therefore conducted a prospective study intended to evaluate pain level in adult patients undergoing BMA at the sternal or iliac crest site to identify factors associated with pain. We enrolled a total of 448 patients who underwent 461 BMA and asked those patients to score their pain intensity after BMA using numerical pain rating scale (NPRS). The following factors: level of anxiety, quality of the information given to the patient, operator’s experience, and bone texture were recorded using a standardized questionnaire. The median NPRS score was 3.5 (IQR [2.0; 5.0]) the sternal site (n = 405) was associated with an increased median NPRS score (3.5 [2.0; 5.0]) compared to the iliac crest (n = 56, 2.5 [1.0; 4.0]; p<0.0001). For those patients who underwent sternal BMA, the median NPRS score was significantly lower when using lidocaine infiltration (p = 0.0159) as compared with no anesthetic use. Additionally there was no significant effect of anesthetic cream found. After multivariate analysis, the model of NPRS score at the sternal site included patient anxiety (p<0.0001) and the use of lidocaine infiltration (0.0378). This study underlines the usefulness of a comprehensive management including pain relief and efforts to reduce anxiety including appropriate information given to the patient during BMA.

## Introduction

Bone marrow aspiration (BMA) allowing quantitative and qualitative assessment of hematopoiesis is essential to the diagnosis, staging, prognosis, follow-up and response to treatment of numerous hematologic and some non-hematologic diseases [1]. In adults, BMA is a well-standardized procedure, which is most often carried out without general anesthesia [1–2]. It is thought to be a safe procedure with 0.07% adverse events according to an English national survey conducted more than ten years ago [3]. Bleeding is considered the most common and most serious adverse event however pain assessment and its management have been poorly documented [4–9]. In numerous countries, the posterior superior iliac crest is generally the preferred site for BMA, which is often followed by a BM biopsy [1,3,10]. In France, when only an aspirate is needed, the most common site of aspiration used in adults is the sternal manubrium even though it can be considered a higher risk procedure [7]. BMA from the iliac crest followed by BM biopsy are rarely performed as first line tests in our practice except for some indications. Sternal manubrium is a more accessible site than the iliac crest, especially in older, immobile or very obese patients and it easily allows applying pressure to the wound to ensure adequate hemostasis [1]. Moreover, it is safely feasible in patients receiving anticoagulant treatment (heparin derivative, vitamin K antagonist with INR within the therapeutic range, or direct oral anticoagulant) if proper procedures are followed. Whatever the site of aspiration, BMA is still considered a painful procedure and standardization of pain prevention remains a major issue. Little however is known regarding the prevalence, severity and predictors of BMA related pain in adults. Most data concern pain evaluation in pediatrics, or in adults undergoing BMA mostly followed by BM biopsy [5,7,11–13]. Many procedures for anesthesia before BMA have been proposed but there are no consensus guidelines that have been provided thus far, considering the lack of prospective studies [9]. Pain combines various individual factors including socio-demographic factors (sex, age, education level, ethnicity), physical and psychological factors, or tumor process [12]. When performing BMA in adults, limiting pain and anxiety is therefore crucial.

In order to optimize the management of patients undergoing BMA, a prospective study was conducted, aiming at i/ assessing pain level during BMA using numerical pain rating scale (NPRS), and ii/ identifying factors associated with lower pain using a standardized questionnaire completed at the end of the BMA procedure. Finally, a model predicting NPRS score in patients with BMA from the sternal site was created.

## Materials and methods

### Study design and data collection

**Fig 1:**
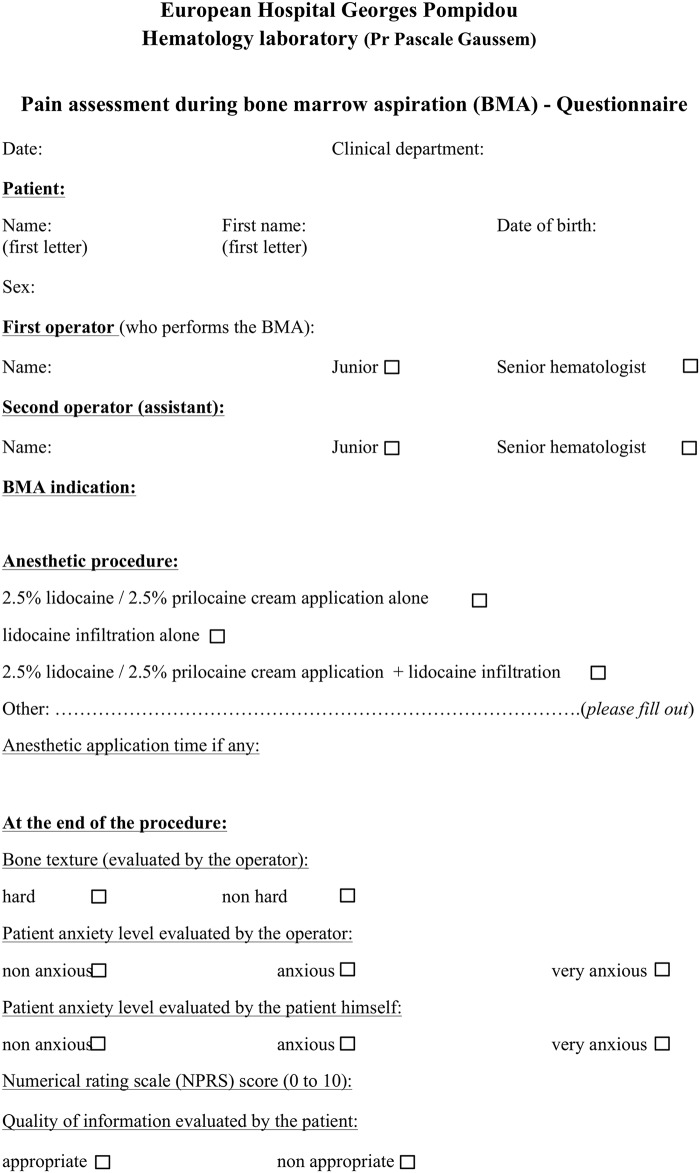
Questionnaire on pain assessment during bone marrow aspiration (BMA).

The study was prospectively conducted in patients undergoing BMA at the university Hôpital Européen Georges Pompidou (HEGP, Assistance Publique-Hôpitaux de Paris-AP-HP, France) between August 2010 and December 2015. HEGP comprises various intensive and acute care units, with no dedicated clinical hematology department. Patients were eligible if they were ≥18 years old and gave their oral consent for participating in the study, and if BMA (without associated BM biopsy) was carried out in the presence of two operators from the hospital Hematology Laboratory, including a senior experienced hematologist. The operator performing the BMA and the assistant could alternatively be either a senior hematologist with an experience of at least 15 years or a junior hematologist (resident). Patients with severe cognitive disorders, deep sedation or language barrier were excluded from the analysis. Within the few minutes following the BMA, a standardized questionnaire was systematically filled out by the assistant (Fig 1). The following data were recorded: demographic patient characteristics (age, sex, clinical department), BMA indication, aspiration site, local anesthesia procedure (see below) and the bone texture evaluated by the operator. The patient was asked to assess pain intensity related to the procedure using a 10-point NPRS for which a score of 0 indicates no pain and a score of 10 indicates the worst imaginable pain. Patients were classified as non-anxious, anxious or very anxious, both according to themselves and by the operator. Patients rated the quality of the information received during the procedure as appropriate or non-appropriate. The Hôpital Européen Georges Pompidou review board provided ethical oversight and study approval. The study was performed in accordance with the Declaration of Helsinki. This research on human subjects was authorized by the French Data Protection Agency (CNIL-1922081).

The primary objective of the study was to evaluate pain level using NPRS in adult patients undergoing BMA at the sternal or iliac crest. The second objectives were to identify factors associated with pain during BMA and to build a model predicting NPRS score in these patients.

### Topical anesthesia procedures

The choice of the topical anesthesia procedure was left to the operator’s discretion. Different anesthesia procedures were proposed: 2.5% lidocaine/2.5% prilocaine cream application (referred below as “anesthetic cream”) at the site of needle insertion (the time of application was recorded); local 20 mg/mL lidocaine infiltration (5 mL at sternal site, 10 mL at crest iliac site); 50% nitrous oxide/oxygen gas premix inhalation. A combination of these procedures could be used. Premedication with an anxiolytic or an analgesic were also recorded.

### Standard operating BMA procedure

The BMA was performed according to a local standard written procedure according to the recommendations of the French Society of Hematology [7]. In France, BMA from the sternal manubrium is preferred in adults, but BMA from posterior or anterior superior iliac crests is also carried out, especially in case of contraindications at the sternal site (sternotomy, chest radiation treatments) or if an associated BM biopsy is needed. Appropriate needles with different length were used according to the aspiration site: 1.6 AWG/20 mm including a guard (Thiebaud Biomedical Device, Margencel, France) for the sternal site, 1.6 AWG/30 mm or 50 mm (Thiebaud Biomedical Device) for the iliac crest. Either the junior or senior hematologist carried out the BMA, assisted by the second operator who immediately spread BM films and filled tubes for supplementary tests if needed (cytogenetic, molecular analysis, immunophenotyping). Explanation was given by the operator to the patient throughout the BMA procedure. With respect for asepsis, the needle was carefully inserted into the bone, the mandrel was removed and a 20 mL syringe was adapted so that a small amount of marrow fluid was aspired to prepare BM films; a second aspiration could be performed using a second syringe in the case of additional tests. Finally, the needle was removed and pressure was applied at the aspiration site to prevent bleeding.

### Statistical analysis

Computations were performed using the R software (version 3.5.1; R Foundation for Statistical Computing, Vienna, Austria). Results were expressed as medians (interquartile range–IQR). All analyses were performed with a linear mixed effect model, using the operator as a random effect, to take into account the potential differences between operators. Tests were done using asymptotic likelihood ratio tests. Since pain score data are not Gaussian, confidence intervals on the model coefficients were obtained using bootstrap to avoid this hypothesis. Covariates were added one by one (“univariate analysis”); anesthetic procedure as well as other covariates with *p* values less than 0.15 were entered into a multiple linear regression model and the covariates with a non-null coefficient, according to the 95% bootstrapped confidence interval (CI), were retained in the final model.

## Results

### Patient characteristics

**Fig 2:**
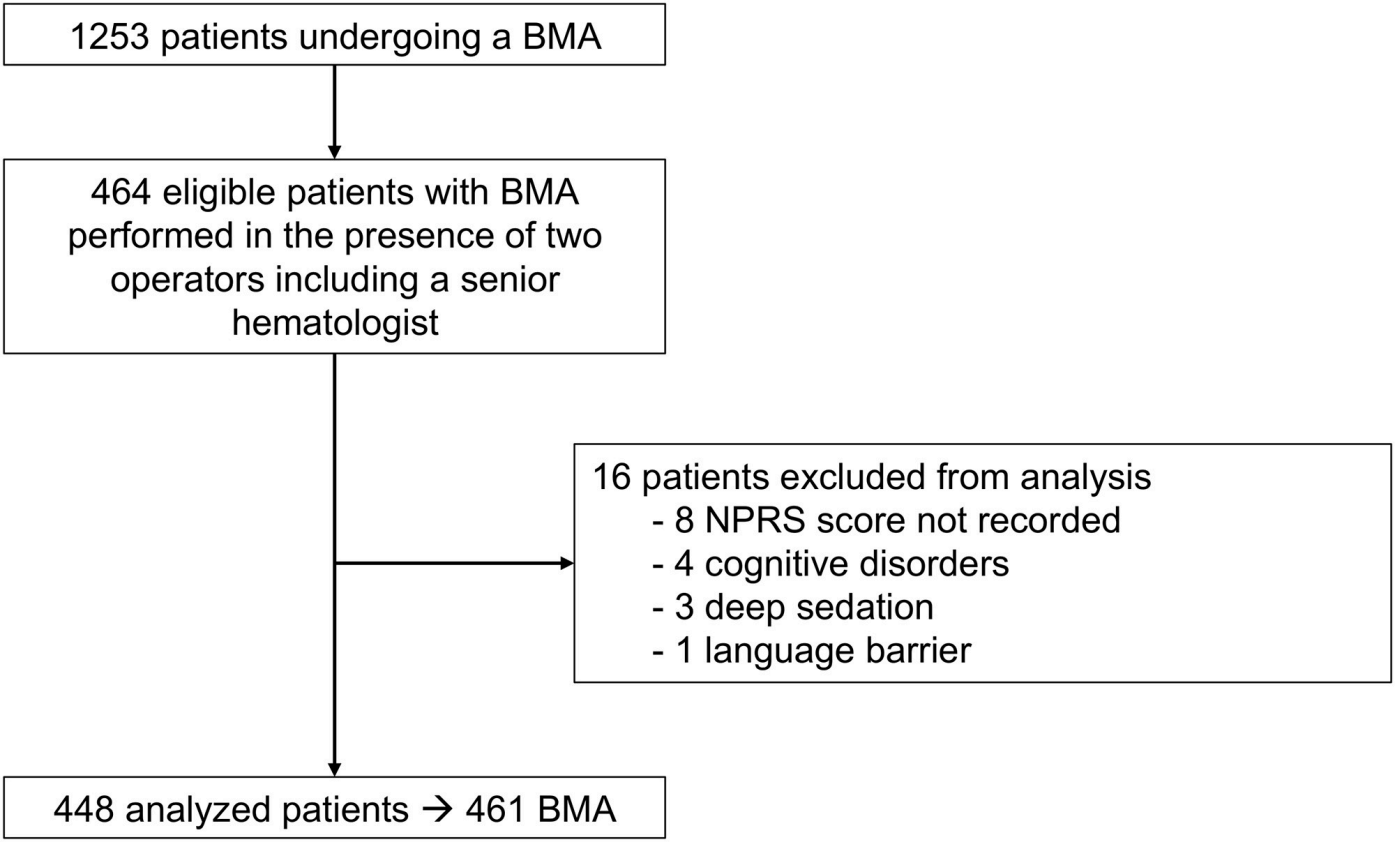
Patient flowchart. BMA: bone marrow aspiration; NPRS: numerical pain rating scale score.

Of the 1253 patients who had undergone BMA at HEGP between August 2010 and December 2015, 464 patients were eligible in the study with a BMA being performed in the presence of two operators including one among the five senior experienced hematologists (Fig 2). The standardized questionnaire could not be completed for 16 patients because of cognitive disorders, deep sedation, or language barrier and these patients were excluded from the analysis. Finally, 448 patients corresponding to 461 BMA were analyzed. Patient characteristics are presented in Table 1. Briefly, the median age was 70 years. More than half of the BMA were carried out in patients from the internal medicine department (30.6%), from the nephrology department (16.1%) or were hematology outpatients (16.1%). Regarding the indication: 141 BMA (30.7%) were performed to explore a monoclonal gammopathy, 98 (21.3%) an isolated non-regenerative anemia, and 80 (17.4%) a bicytopenia or a pancytopenia. No complications related to the BMA procedure were recorded.

**Table 1:** Patient characteristics and bone marrow aspiration indication.

**Patient characteristics**	
Number of patients	448
Age, median (range)	70 (IQR 59; 81)
Sex, n (%)	
Males	261 (58.3)
Females	187 (41.7)
**Clinical department, n (%)**	
Internal medicine	141 (30.5)
Hematology outpatients	74 (16.1)
Nephrology	74 (16.1)
Geriatrics	41 (8.9)
Surgery units*	17 (3.7)
Intensive care unit and emergency unit	16 (3.5)
Oncology	8 (1.7)
Other departments**	90 (19.5)
**BMA indication, n (%)**	
Monoclonal gammopathy	141 (30.7)
Isolated non-regenerative anemia	97 (21.0)
Agranulocytosis	8 (1.7)
Thrombocytopenia	48 (10.4)
Bicytopenia or pancytopenia	80 (17.4)
Lymphoproliferative disorders	15 (3.3)
Myeloproliferative neoplasms	25 (5.4)
MDS/MPN	14 (3.0)
Acute leukemia	17 (3.7)
Hemophagocytosis, histiocytosis	7 (1.5)
Metastatic tumors	7 (1.5)
Myeloculture	2 (0.4)
**Total BMA**	**461 (100)**

BMA: Bone marrow aspiration; IQR: interquartile range; MDS/MPN: myelodysplastic syndromes/myeloproliferative neoplasms;

*Surgery units: orthopedic and urologic surgery units;

**Other departments: cardiology, gastroenterology, pulmonary, immunology and vascular medicine departments

### BMA characteristics, anxiety and quality of information

Overall, 405 BMA (87.9%) were performed from the sternal site and 56 (12.1%) from the iliac crest site, reflecting the French guidelines of BMA procedure (Table 2). None of the BMA at the iliac crest site was followed by a BM biopsy. The vast majority of BMA (n = 441, 95.7%) were performed under anesthesia as shown in Table 2; only 20 BMA (4.3%), of which 19 from the sternal site, were performed with no anesthesia. The most commonly used anesthesia procedure for sternal BMA was the application of anesthetic cream alone (n = 336, 83.0%) with a median application time of 60 min [60; 90]. In contrast, the most common anesthetic procedure for iliac crest BMA was lidocaine infiltration alone (n = 49, 87.5%). Combined anesthetic procedures have been performed in a minority of patients: five of the 461 BMA were conducted after 50% nitrous oxide/oxygen gas premix inhalation, three patients had received either an anxiolytic or a morphine based analgesic before BMA.

**Table 2:** NPRS scores according to the site and the anesthetic procedure.

BMA site	Sternal site		Iliac crest site	
n (%)	405 (87.9)		56 (12.1)	
Anesthetic procedure	n (%)	NPRS score Median [IQR]	n (%)	NPRS score Median [IQR]
No anesthesia	19 (4.7)	5.0 [3.9–6.0]	1 (1,8)	3.8 (NA)
Anesthetic cream alone	336 (83.0)	3.5 [2.0–5.1]	2 (3.6)	2.6 (NA)
Lidocaine infiltration alone	18 (4.4)	3.2 [1.6–4.5]	49 (87.4)	2.40 [1.0–4.0]
Anesthetic cream + lidocaine infiltration	28 (6.9)	2.8 [1.6–4.0]	3 (5.4)	1.5 (NA)
Others*	4 (1.0)	2.8 [1.8–3.6]	1 (1.8)	4 (NA)

NPRS: numerical pain rating scale; BMA for bone marrow aspiration; IQR for interquartile range; NA non-available.

*Others: 50% nitrous oxide/oxygen gas premix inhalation associated with lidocaine infiltration or anesthetic patch.

Of the 461 BMA, 335 (72.7%) were performed by a senior hematologist and 111 (24.1%) by a junior, with missing data for 15 BMA (3.3%). The patients reported to be very anxious for 99 BMA (21.5%), anxious for 147 (31.9%) and non-anxious for 211 (45.8%, missing data for 4 BMA). Interestingly, a good agreement was observed when the anxiety level was assessed by the operator with 77.4% of perfect agreement, and only 3 cases of strongly discordant evaluation. After 425 of the 461 BMA (92.2%), patients considered that they had been well informed about the procedure. In 17.1% of BMA, bone texture was evaluated as hard by the operator.

### NPRS and factors potentially influencing NPRS

For all BMA (n = 461), the median NPRS score was 3.5 IQR [2.0; 5.0]. During the study period, the vast majority of patients experienced BMA for the first time with only 12 (2.7%) patients having experienced several BMAs (11 with two BMA and one with three). One third of these repeated BMA experienced lower pain level, one-third equal pain and one-third a higher pain level.

**Fig 3:**
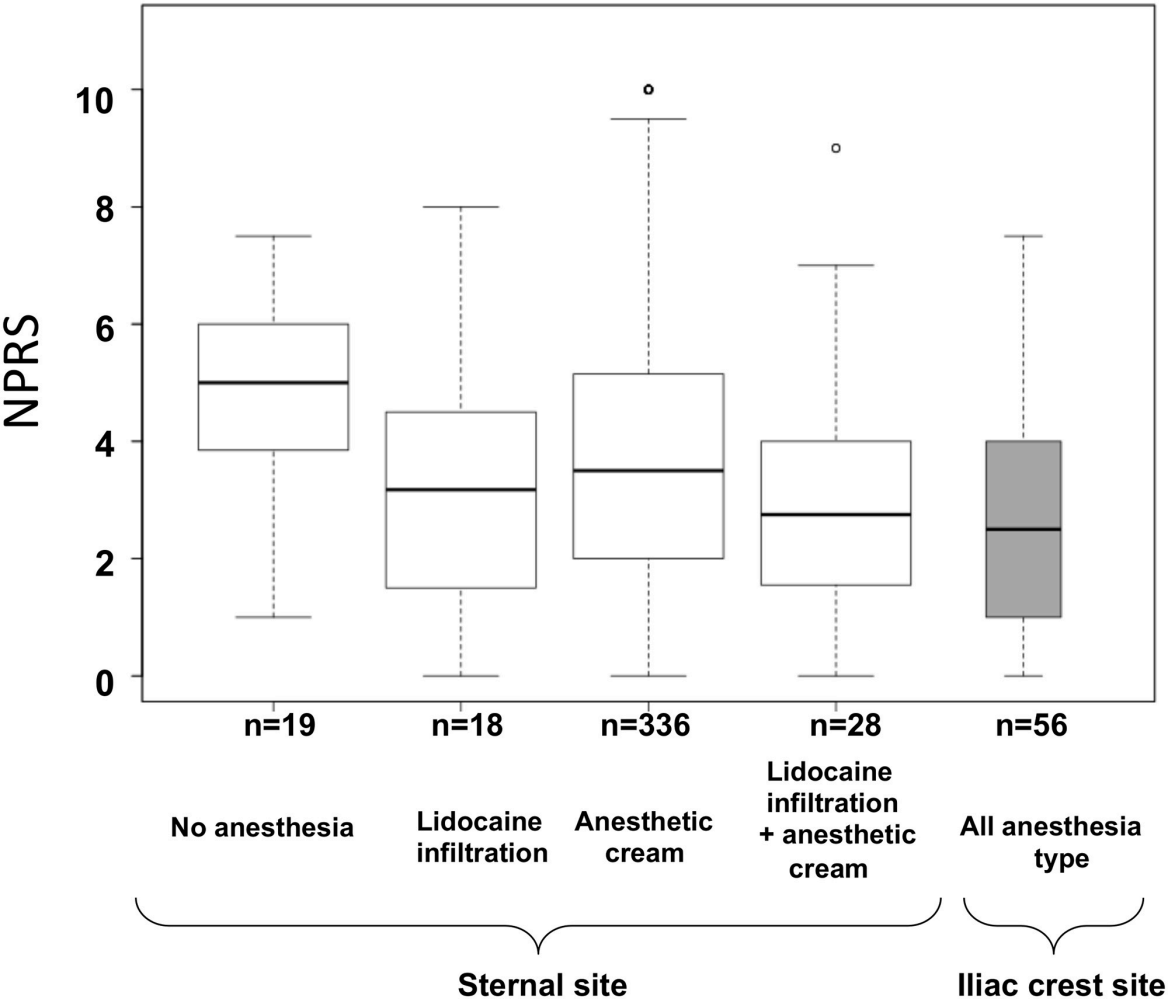
NPRS score according to BMA site and the anesthetic procedure. Boxplots represent median values (horizontal line) and whiskers the 5^th^ and 95^th^ percentiles. BMA: bone marrow aspiration; NPRS: numerical pain rating scale score.

For subsequent univariate analyses, all BMA were used. The sternal site was significantly associated with an increased pain when compared to iliac crest, with median NPRS scores of 3.5 [2.0; 5.0] and 2.5 [1.0; 4.0], respectively (p < 0.0001, Fig 3). Focusing on BMA at sternal site (n = 405), we found that lidocaine infiltration was associated with a lower NPRS score compared with the absence of anesthesia (NPRS: −1.06 point in average; 95% CI [−1.92; −0.20]; p = 0.0158; Fig 3); there was no significant effect of the anesthetic cream compared to no anesthesia used (NPRS: −0.46 point; 95% CI [−1.35; +0.43]; p = 0.3125). No synergic, nor antagonist effect was found when using both methods (p = 0.9498, test of interaction).

**Fig 4:**
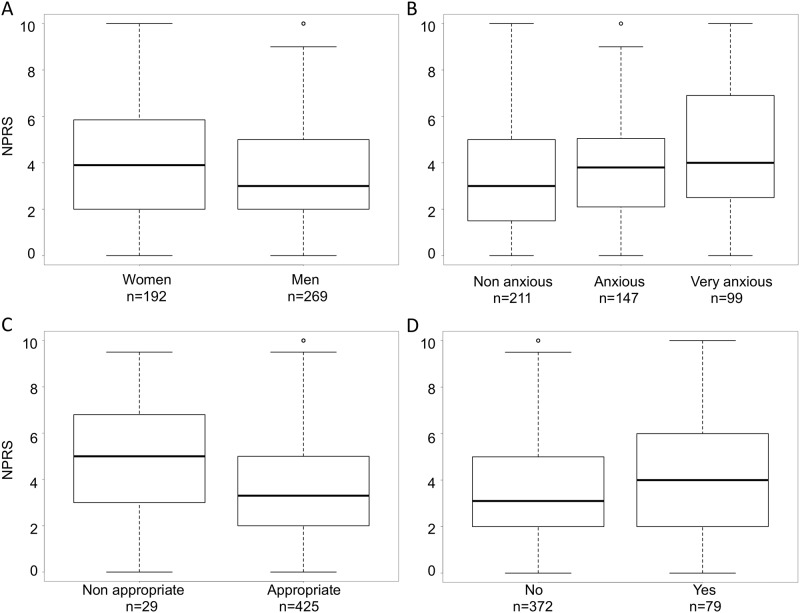
Factors influencing NRPS score during BMA (sternal and iliac crest site). Effect on sex (A), anxiety evaluated by the patient (B), quality of information received during BMA procedure (C) and bone texture (D) on NPRS score. Boxplots represent median values (horizontal line) and whiskers the 5^th^ and 95^th^ percentiles.

Among individual factors potentially influencing NPRS, we found that BMA was significantly more painful in women than in men (p = 0.0352) (Fig 4A). In addition, NPRS score significantly increased with anxiety assessed either by the patient himself (p<0.0001, Fig 4B) or the operator (p<0.0001, Table 3). Well-informed patients had a significantly lower NPRS score than those who were not well informed (p = 0.0233, Fig 4C). Finally, there was a trend for higher NPRS score when bone texture was evaluated as hard versus not hard (p = 0.0582, Fig 4D). Among other variables tested, *i*.*e*. age, BMA indication, duration of anesthetic cream application, experience of the operator (senior/junior), identity of the operator, none were found significantly associated with NPRS in univariate analyses.

**Table 3:** NPRS scores in the whole cohort (n = 461) according to patient’s anxiety assessed by the operator or by the patient himself, and according to the quality of information.

n (%)		NPRS score median [IQR] (min-max)
**Patient anxiety assessed by the operator**		
Very anxious	89 (19.3)	5 [2.9; 7.0] (0–10)
Anxious	155 (33.6)	4 [2.0; 5.4] (0–10)
Non-anxious	205 (44.5)	3 [1.5; 4.0] (0–10)
Missing data	12 (2.6)	NA
**Patient anxiety assessed by the patient himself**		
Very anxious	99 (21.4)	4.0 [2.5; 6.9] (0–10)
Anxious	147 (31.9)	3.8 [2.1; 5.1] (0–10)
Non anxious	211 (45.8)	3.0 [1.5; 5.0] (0–10)
Missing data	4 (0.9)	NA
**Quality of information given to the patient**		
Appropriate	425 (92.2)	3.3 [2.0; 5.0] (0–10)
Non appropriate	29 (6.3)	5.0 [3.0 6.8] (0–9.5)
Missing data	7 (1.5)	NA

NPRS for numerical rating scale score; IQR for interquartile range; NA non-available.

### Modeling NPRS score for sternal BMA

We sought to build a model of the NPRS score for sternal BMA, representing 87.9% of BMA performed in the present cohort. This model included variables potentially influencing NPRS as shown in Table 4. Operator-evaluated anxiety was not included, since it was strongly concordant with patient-evaluated anxiety. In the final model, only the use of lidocaine infiltration and patient anxiety were kept, with an inter-operator variability of around 0.53 point and an inter-patient variability of around 2.34 points. The NPRS score could be predicted by the formula (Table 4):

NPRSscore=3.64−1.06iflidocaineinfiltration+(1.025ifanxiousor1.39ifveryanxious).

**Table 4:** Regression model of the NPRS score after sternal BMA.

Variable	*Linear regression—Univariate analysis*			*Multivariate analysis*		
	βCoefficient	95% CI	*p-value*	βCoefficient	95% CI	*p-value*
Sex (reference female)						
-male	−0.53	[−1.02; −0.05]	0.0315	−0.46	[−0.95; 0.029]	0.0650
Anesthetic procedure (reference: none)						
- lidocaine infiltration alone	−1.10	[−2.69; 0.48]	0.1618	−1.06	[−2.65; 0.53]	**0.0378**
- anaesthetic cream alone	−0.47	[−1.61; 0.65]		−0.26	[−1.44; 0.93]	0.824
- lidocaine + anesthetic cream	−1.52	[−2.98; −0.07]		−0.07	[−1,90; 1,77]	0.9434
- other	−1.62	[−4.28; 1.00]		−0.89	[−3.78; 1,99]	0.5439
Anxiety (reference: non anxious)*						
- anxious	0.92	[0.38; 1.46]	< 0.0001	0.97	[0.42; 1.52]	**< 0.0001**
- very anxious	1.43	[0.83; 2.02]		1.31	[0.71; 1.91]	
Information (reference: non appropriate)						
- appropriate	−1.04	[−2.07; −0.01]	0.0488	−0.99	[−2.00; 0.01]	0.0529
Bone texture (reference: not hard)						
-hard	0.52	[−0.12; 1.15]	0.1095	0.57	[−0.05; 1.20]	0.0709
Operator’s experience (reference: junior)	0.34	[−0.43; 1.11]	0.370	Not retained for multivariate analysis		
-senior						

*According to the patient

Noteworthy, the patient’s sex, the quality of information and the bone texture were not retained in the final model; in these cases, p values were comprised between 0.05 and 0.08.

## Discussion

To our knowledge, the present study is the first one assessing pain using NPRS during BMA performed alone by identifying factors influencing pain and their respective impact on the NPRS. Which lead us to the building of a model for evaluating pain at the sternal site. Overall, we found a median NPRS score of 3.5 in the entire cohort (n = 461 BMA). In some studies, pain experienced from the BMA was recorded at different steps, *i*.*e*. during the infiltration of soft tissue and bone with lidocaine, the needle insertion itself and lastly during BM aspiration [13]. Here, we chose to ask patients to grade the pain and their anxiety during the global procedure. In the literature, median NPRS score varied between 1.9 and 5.0 [14], reflecting the heterogeneity of practices and studies. Indeed, different patient settings were evaluated, with most studies focusing on hematology patients who often experience multiple BMA [15]. In contrast to these studies, most of patients evaluated experienced BMA for the first time. Most studies in the literature were conducted in patients experiencing BMA from iliac site followed by BM biopsy, after lidocaine infiltration with little specific data regarding BMA alone. We provide here for the first time pain experienced during BMA from both sternal and iliac site, using different anesthetic procedures for the sternal site. BMA from the iliac crest site resulted in a significantly lower pain than from the sternal site; however making direct comparisons between sites are debatable given the heterogeneity of anesthetic procedures related to sternal site in our study. For patients who had undergone a BMA from the sternal site, we found that lidocaine infiltration with or without the anesthetic cream resulted in the lowest NPRS compared with no anesthesia. Furthermore, lidocaine infiltration significantly contributed to a lower pain in our final model predicting NPRS. Therefore, our study confirms that aspiration from the sternal site might be an alternative to BMA from the iliac crest site with regard to pain level, provided that lidocaine infiltration is performed.

Anxiety was also found to be a main predictor of pain during the procedure confirming that the perception of pain may be influenced by the psychological state of the patient. In our cohort, more than half of the patients were anxious or very anxious before BMA. Therefore, prevention and management of pain may require the association of pharmacological and non-pharmacological interventions to reduce pain related to aspiration and that related to anticipatory anxiety in agreement with previous studies [4–5]. It has been shown that a prior negative experience of BMA can lead to fear and anxiety for any future BMA and has been associated with a more painful procedure [4]. Reducing patient’s anxiety during the first BMA is thus challenging, especially in hematology patients.

Beside the use of lidocaine and anxiety as factors significantly influencing pain, we identified other factors that could have an impact on NPRS, although not retained in the final model regarding pain at the sternal site. Women had a trended to have a higher NPRS score. In the literature, inconsistent results have been shown regarding the influence of age and sex on pain level during BMA [4,16–17]; again, this can be explained by the heterogeneity of patient settings.

We also found that the quality of the information during the procedure could decrease pain. Similarly, Degen *et al* found that an appropriate explanation of the procedure in a comprehensive and didactic way allowed a trust bond to be formed between the physician and the patient: it was essential to ensure patient confidence and was associated with less pain during iliac BMA [4–5]. In the present study, 93.6% of patients considered themselves well informed. Since appropriate information regarding the procedure might decrease pain level, there is no doubt that BMA operators should make an effort to properly explain the procedure. The patient should be warned of the possibility of aspiration pain before it occurs and be reassured that it will be brief, since anesthesia has no effect on this pain. In our study, we observed that the hard texture of bone could be associated with increased pain, under this circumstance we know by experience that the BMA takes more time. We did not find any relationship between the experience of the operator and the pain felt, as previously published [9].

One strength of our study is the prospective study design with the use of a standardized questionnaire fulfilled in the presence of a senior experienced hematologist during the procedure, therefore limiting biases. Noteworthy, the questionnaire was fulfilled by the assistant, and not by the operator who performed the BMA, to obtain an answer from the patient in a more objective way was moved down just before our limitations of the study.

Our study has some limitations. Firstly, our study was only conducted in patients undergoing BMA in the presence of an experienced senior hematologist, *i*.*e*. in 38% of the patients undergoing BMA during the study period. This was chosen to minimize biases due the potential operator dependent variability conducting the study. Secondly, this work was a mono-centric study and evaluating mostly BMA from the sternal site raises the question of whether our findings could be generalizable to other centers. We know that in many countries, the choice of the sternal site is thought to be more hazardous than iliac crest. However, most fatal adverse events that occurred during BMA from sternal site were performed by physicians with lack of experience or performing BMA at an incorrect aspiration site [18]. Remarkably, we did not report any adverse effects after sternal or iliac BMA in our study. Regarding the aspiration site, the sternum is close to the skin whereas the iliac crest is deeper under a panicle. The question that arises is how the sternal site easily reachable is more painful. One can hypothesize that, as for blood sampling, the sight of the needle might play a significant role in pain perception. Therefore, if the patient is very anxious or will be at risk of a more painful procedure, iliac crest site should be chosen over sternal site. Third, this study was exploratory, hence we could not predefine a sample size. To confirm our main result, regarding the benefit of the use of lidocaine infiltration versus the use of anesthetic cream alone, a validation study should be performed, ideally randomizing patients in two groups of pre-specified size. Finally, we built a model explaining NPRS score at the sternal site, including both the use of lidocaine infiltration and anxiety level. The next step will be to prospectively validate this model in external cohorts and other settings.

## Conclusion

Our study suggests that for BMA from any site, the patient needs to be reassured and well informed regarding the procedure in an attempt at the lowering pain level experienced. For BMA from the sternal site, we recommend the use of lidocaine infiltration. Additional procedures like hypnosis or music therapy could improve the psychological management of the procedure and remain to be investigated [19].

## Supporting information

S1 Table Patient data collection. (XLSX)
